# The progression of heartworm associated respiratory disease (HARD) in SPF cats 18 months after *Dirofilaria immitis* infection

**DOI:** 10.1186/s13071-017-2425-9

**Published:** 2017-11-09

**Authors:** A. Ray Dillon, Bryon L. Blagburn, Michael Tillson, William Brawner, Betsy Welles, Calvin Johnson, Russell Cattley, Pat Rynders, Sharron Barney

**Affiliations:** 1 College of Veterinary Medicine, Auburn University, Auburn, Alabama 36849 USA; 2 Department of Clinical Sciences, College of Veterinary Medicine, Auburn University, 1220 Wire Road, Auburn, Alabama 36849 USA

**Keywords:** Feline, Heartworm, *Dirofilaria immitis*, Respiratory disease, HARD

## Abstract

**Background:**

Heartworm-associated respiratory disease (HARD) in cats is induced by the arrival and death of immature adult *Dirofilaria immitis* in the pulmonary system and is indistinguishable from mature adult heartworm infection.

**Methods:**

A controlled, blind research study investigated the long-term (18 months post infection, PI) consequences of the inflammatory response associated with the death of immature adult heartworms in cats.

Three groups of cats, 10 per group, were infected with 100 third-stage (L3) larvae by subcutaneous injection. Group A cats were treated with selamectin (Revolution®; Zoetis) per label directions at 28 days PI and once monthly for 17 months. Group B cats were treated orally with ivermectin (Ivomec®; Merial) at 150 μg/kg) at 70 days PI, then every 2 weeks for 15 months. Group C cats were untreated PI. At baseline (Day 0) and on Days 70, 110, 168, 240, 309, 380, and 505 PI, peripheral blood, serum, bronchial lavage, and thoracic radiographic images were collected.

**Results:**

The selamectin-treated cats (Group A) and ivermectin-treated cats (Group B) were free of heartworms or heartworm fragments at necropsy. All cats became heartworm antibody positive at some time point in the study except for one cat in Group A. Only cats in Group C (all with adult heartworms) were heartworm antigen positive. The heartworm antibody titer for Group B was highest on Days 110 to 168 and then decreased over time and 50% were serologically antibody negative on Day 240. Eosinophilic bronchoalveolar lavage (BAL) cytology and peripheral eosinophilia were most pronounced on Day 110 in all cats. Randomly distributed myofibrocytes in the lungs of some Group A cats suggest that precardiac larval stages were affecting the lungs. Radiographs in Group B cats demonstrated partial resolution of the initial HARD reaction but chronic myofibrocyte proliferation was histologically evident 18 months after infection.

**Conclusion:**

HARD was induced by immature adult worm infection with progressive improvement starting 6 to 8 months after infection but histologic lesions were evident in some cats 18 months after infection. The serologic antibody assay was negative in 50% of cats at 8 months and 100% of cats at 18 months post infection. Abnormal radiographic lung patterns continued in a subset of Group B cats for months after heartworm antibody serology and BAL cytology returned to normal.

## Background

Heartworm-Associated Respiratory Disease (HARD) is induced by the arrival, at 70 to 90 days post infection (PI), and death of immature adult *Dirofilaria immitis* and without subsequent development of mature adult heartworms (HWs) in cats [1, 2]. In cats observed for 8 months, the lung disease consists of bronchial, interstitial, and pulmonary arterial disease. After a one-time infection, cats with HARD develop an initial positive serologic HW antibody titer and radiographic lung disease that cannot be distinguished from cats with mature adult HW infection. An eosinophilic cytology on examination of bronchoalveolar lavage (BAL) fluid is observed in both cats with HARD and cats with mature adult infections. Cats with HARD continued to have radiographic lesions consistent with HW disease but 50% were HW antibody negative and had no adults on necropsy at 8 months PI [2]. Unfortunately, a mistaken inference has been that cats that are infected with *D. immitis* and seroconvert from positive to negative represent “self-cure” with no clinical consequences [3, 4]. Cats become HW antibody positive before the immature adult stages enter the heart, but the clinical importance of this stage has not been investigated [5–9].

Development of HARD as a consequence of immature adult HWs only is consistent with a clinical study of 210 cats in private practices in which 30% of cats with clinical signs and radiographs consistent with HARD seroconverted to negative antibody titers on recheck examinations 3 month after presentation [10–12]. Further, the concept of HARD associated only with immature adult infections is supported by a study of random-sourced cats at necropsy, which demonstrated cats that had pulmonary artery lesions and were serologically positive, but had no evidence of adult HWs at necropsy [13].

The purpose of this study was to evaluate the clinical progression and consequences of HARD 18 months after infection. Death of immature adult HWs is associated with an intense inflammatory bronchial, interstitial, and pulmonary arterial reaction which we hypothesized would attenuate with time after infection but would create a chronic proliferative infiltration of the interstitium, peribronchial, and periarteriolar areas of lung.

## Methods

### Animal model

To determine the long-term consequences associated with immature HW death, three groups (*n* = 10) of specific pathogen–free (SPF) neutered male and spayed female 6-month-old cats were utilized (Groups A, B, and C). All cats were infected with 100 L3 larvae (Missouri strain) by subcutaneous (SQ) injection into the flank and observed for 510 days.

Group A cats served as controls for the absence of adult HWs. After infection, the cats in Group A were treated with topically applied selamectin (Revolution®, Zoetis), dosage based on body weight and weight range as indicated by label, once a month beginning 28 days post infection (PI), to kill the immature larvae before they reach the pulmonary arteries.

Group B cats were infected and the larvae were allowed to mature for 70 days. At 70 days PI, ivermectin (Ivomec®; Merial) 150 μg/kg per os, was administered every 2 weeks for 16 months.

Group C cats were infected with L3 larvae and untreated to allowed development of mature adult HWs.

All groups of cats were housed as isolated groups in the indoor animal rooms of the Laboratory Animal Health Veterinary Research Building at Auburn University to prevent exposure to mosquitoes that might be carrying HW larvae. The protocol was approved by the Auburn University Institutional Animal Care and Use Committee and was conducted in an AAALAC-accredited, environmentally isolated facility.

### Data collection

At baseline (Day 0) and on Days 70, 110, 168, 240, 309, 380, and 505 PI, peripheral blood was collected for complete blood count (CBC) and serum for serology; and on Days 0, 110, 168, 240, 309, 380, and 505 PI radiographic ventrodorsal (VD) and lateral thoracic images were acquired and bronchoalveolar lavage (BAL) performed with 10 mL of lactated Ringers solution under sedation with an intramuscular (IM) dose of medetomidine (Domitor®, Zoetis, NY), butorphanol (Torbugesic®; Zoetis), ketamine (ketamine hydrochloride; Zoetis), and after the procedures an IM dose of atipamezole (Antisedan®; Zoetis) was administered. Cats were monitored daily and physical examination performed weekly.

At the termination of the study (Day 510), cats were humanely euthanized after sedation using pentobarbital sodium and phenytoin sodium solution 1 mL/10 lbs. given intraperitoneally (Euthasol®, Virbac AH). Complete necropsies were conducted with collection of lung, heart, brain, kidney, and liver for histopathology studies. Immediately post mortem a blood sample from the right ventricle was collected for serology. Right caudal lung lobes were fixed perfused with 10% formalin via the bronchi to a pressure of 14 cm H_2_O. Pathologists and radiologists were blinded to the treatment groups to which cats were assigned, creating a controlled, blind study format.

### Data evaluation

Data reported in this paper include results from thoracic radiographs, complete peripheral blood counts, serology, BAL cytology, serology, and lung histopathology.

Evaluation of thoracic radiographs consisted of severity scoring (0–3) for 12 different parameters, including bronchial, parenchymal, vascular, and cardiac changes [10, 14, 15]. Histopathology was based on subjective evaluation of severity (scoring 0–3) of pathology of the right caudal lung lobe [14, 15]. Structures scored by two separate pathologists included pulmonary artery, pulmonary arterioles, bronchi, bronchioles, and alveolus including interstitial smooth muscle proliferation. For the bronchioles, the airways were also measured for morphometric analysis by calculating the area of the lumen, total area of bronchial wall and lumen, and the area of the bronchial wall. A minimum of five bronchioles were measured on digital images for each cat using ImageJ (NIH, http://rsb.info.nih.gov/ij/). Data were analyzed by comparison of the lumen to wall ratio, total area to wall ratio, and total area to lumen ratio. The BAL data (Clinical Pathology Laboratory, Auburn University, College of Veterinary Medicine) were recorded as subjective descriptive narrative and expressed cellular morphology as percentage of cell types compared with nucleated cells observed [14]. Serologic evaluation of batched frozen serum was performed (Antech Diagnostics) with DiroCHECK® (Symbiotics; currently Zoetis) in microwell plates for spectral analysis of HW antigen and enzyme-linked immunosorbent assay (ELISA) for HW antibody.

All statistical analysis of data was performed with Systat Software (Sigma Plot 12, Systat Software Inc). Data points across time within each group were evaluated by paired T testing for changes between collection dates. Data between groups was evaluated with ANOVA and then by pairwise analysis. Pearson’s and Spearman’s correlations were performed for linkage between histologic, radiographic, serologic, hematologic, and BAL results.

## Results

### Necropsy

At day 510 PI, no live HWs or HW fragments were found in any cats in Group A or Group B. All 10 cats in Group C had viable adult HWs or worm fragments (Table 1). One cat in Group C died acutely on Day 168; more than 10 immature and multiple fragments/degenerated worms were noted and the lungs were severely congested. On gross examination, lungs of cats in Groups A and B inflated normally during fixation. Lungs of cats in Group C were not uniformly abnormal and the gross appearance was not predictive of the number of HWs.

**Table 1:** Adult heartworms at necropsy of cats in Group C (infected untreated) on Day 510 PI

Cat number	Viable adult worms^a^ (male, female)	Dead worms or fragments
E189	0 (0, 0)	2 dead
F339	1 (1, 0)	1 dead 2 fragments
G365	3 (0, 3)	5 fragments
G409	1 (1,0)	8 fragments
F362	1 (0, 1)	5 fragments
F370	0 (0, 0)	3 dead
G006	1 (0, 1)	12 fragments
F194	3 (1, 2)	2 fragments
E297	1 (0,1)	1 fragments
F063^b^	> 10 immature	>10 fragments

^a^Viability of adult worms was based on motility in warmed Hanks solution after removal. Fragments are sections of dead adults and not a result of dissection during removal

^b^Cat died acutely on Day 168

### Peripheral CBC

None of the cats developed an inflammatory leukogram during the observation at any time point. An eosinophilia (>1500/μL) was noted most prominently at Day 110 PI in the majority of the cats in Groups B and C and 40% of cats in Group A (Table 2). All cats developed an eosinophilia (>1500/μL) in the infected untreated Group C but the increase was not consistent in each individual cat over time. In the abbreviated infected cats (Group B), eosinophilia, if noted, was not as elevated as in Group C. Basophils were noted in cats in Groups B and C but were not consistently present in the same cat over multiple time points.

**Table 2:** Number of cats (10/group) with peripheral circulating eosinophilia (>1500/μL) after L3 *D. immitis* infection

	Day 0		Day 70		Day 110		Day 168		Day 240		Day 300		Day 390		Day 505	
	> 1500	Range	> 1500	Range	> 1500	Range	> 1500	Range	> 1500	Range	> 1500	Range	> 1500	Range	> 1500	Range
Group A	0	267–1395	2	190–6511	4	241–1915	2	95–1564	0	503–1286	0	178–1064	1	327–1771	0	0–994
Group B	0	317–1450	6	206–14,891	8	757–5594	2	218–3400	4	419–1654	1	228–1600	1	338–1828	0	0–1105
Group C	0	87–1400	3	137–5880	7	137–2025	7	519–8831	8	586–7454	6	941–6334	8	831–10,620	7	184–5264

Group A: selamectin topical initiated on Day 28 PI; Group B: oral ivermectin initiated on Day 70 PI; Group C: infected, no treatment (9 cats in group Day 240–505)

### Bronchoalveolar lavage

The BAL cytology was expressed as percentage of nucleated cells as grade 0–3 (0 < 16%, 1 = 17–35%, 2 = 36–60%, 3 ≥ 61%) [7]. Group B cats demonstrated the most eosinophilic BAL cytology on Day 110 but results were inconsistent in individual cats after Day 168 (Table 3). Some Group A cats had an eosinophilic BAL cytology even when HW antibody results were negative. All cats in Group C developed a grade 1 eosinophilic cytology at some time point over the 510-day observation period but it was not consistent in the individual cats over time. No correlation between BAL eosinophilic cytology results, and peripheral blood eosinophilia was noted when cats were evaluated by group or when data from all 30 cats were combined. There was no correlation between the presence of basophils on CBC and BAL eosinophil cytology.

**Table 3:** The number of cats in each group^a^ with bronchoalveolar lavage eosinophilic cytology after L3 *D. immitis* infection

	Day 0			Day 110			Day 168			Day 240			Day 300			Day 390			Day 505		
Grade^b^	Group			Group			Group			Group			Group			Group			Group		
	A	B	C	A	B	C	A	B	C	A	B	C	A	B	C	A	B	C	A	B	C
0	7	10	10	9	5	2	5	9	6	4	10	3	5	7	3	5	7	2	7	10	3
1	2	0	0	1	0	1	3	1	2	1	0	1	3	1	1	1	1	2	3	0	2
2	1	0	0	0	4	3	2	0	0	2	0	2	1	1	1	2	0	1	0	0	3
3	0	0	0	0	1	4	0	0	2	3	0	3	3	0	4	0	1	4	0	0	1

^a^Group A (10): selamectin topical initiated on Day 28 PI; Group B (10): oral ivermectin initiated on Day 70 PI; Group C (10): infected no treatment

^b^Grade 0 < 16%, Grade 1 = 17–35%, Grade 2 = 36–60%, Grade 3 > 61% (percent represents the number of eosinophils compared to total nucleated cells observed)

### Serology

**Fig. 1:**
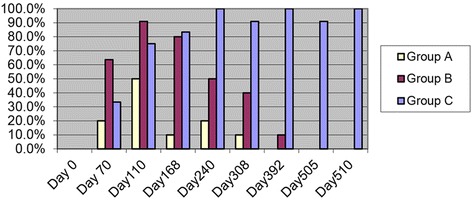
Heartworm antibody titers in serum of SPF cats infected with L3 *D. immitis.* Group A (selamectin at 28 days PI), Group B (ivermectin at 70 days PI), Group C (no treatment). Percent of cats serologically antibody positive after L3 *D. immitis* infection. Day 510 represents right ventricle sample immediately post-mortem

All cats in Groups B and C and 9/10 cats in Group A were positive for HW antibody at one of the time points from Day 70 to Day 510. In Group A, one in 10 cats was still positive on Day 308. More cats in Group B were positive on Day 110 than at any other time point (Fig. 1) and one in 10 was positive on Day 392. On Day 240 and for the duration of the observation, 90 to 100% of cats in Group C were antibody positive.

**Fig. 2:**
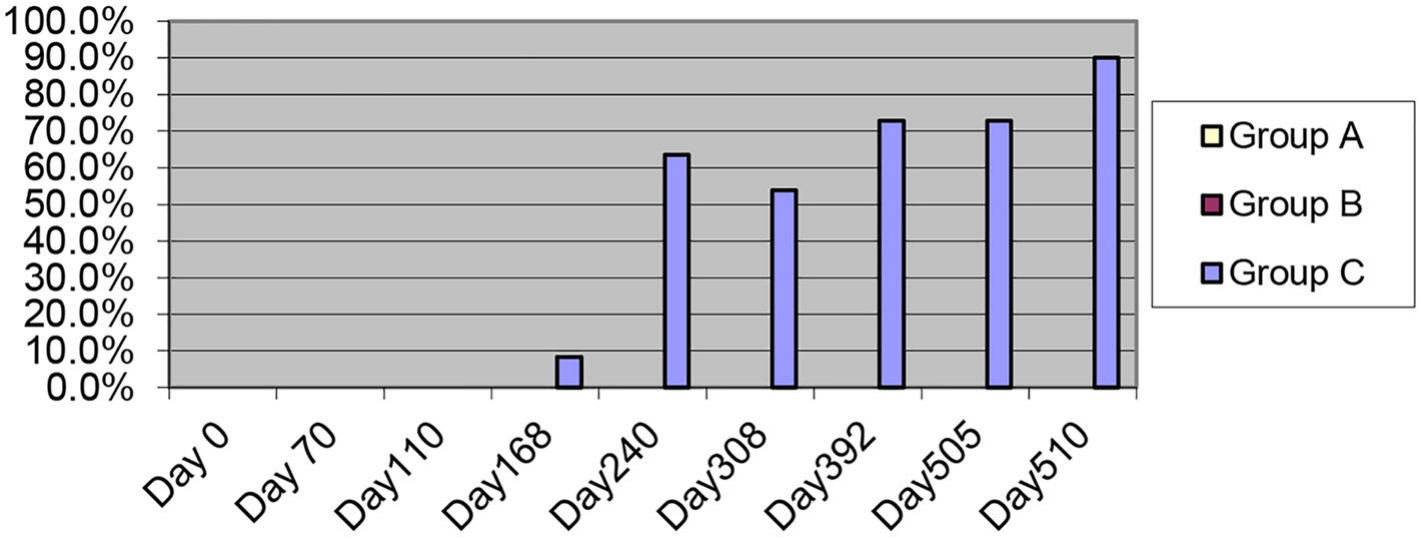
Heartworm antigen assay in SPF cats infected with L3 *D. immitis*. Group A (selamectin at 28 days PI), Group B (ivermectin at 70 days PI), Group C (no treatment). Percent of cats serologically antigen positive after L3 D. immitis infection. Day 510 represents right ventricle sample immediately post-mortem

None of the cats in Group A or B were HW antigen positive at any time point. On peripheral blood serology, all of the cats in Group C except for one was antigen positive at one of the time points (Fig. 2). Comparing peripheral blood samples to direct right ventricular samples immediately post mortem on Day 510 compared with Day 505 resulted in an increase in percentage of cats that were antigen positive. The optical density on spectral analysis for individual cats, however, was increased in four cats and decreased in five cats.

### Radiology

**Fig. 3:**
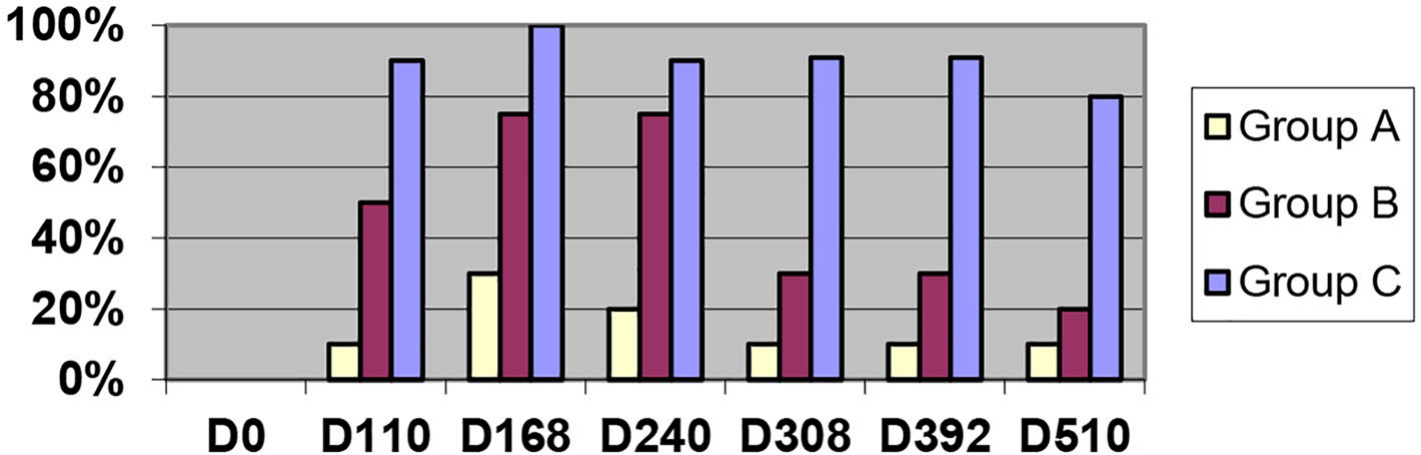
Percentage of cats with radiographic bronchial-interstitial score of >2 (scale 0–3) after *D. immitis* infection. Group A (10) Selamectin topical initiated day 28 pi, Group B (10) Oral ivermectin initiated day 70 pi, Group C (10) no treatment

Cats in Group A generally had normal radiographs throughout the study with the exception of three cats with bronchial/interstitial pattern identified at Day 168 which decreased over time (Fig. 3). The pattern identified was subtle and distributed focally in the caudal lobes. One cat in Group A had an enlarged caudal pulmonary artery (Days 168–510).

**Fig. 4:**
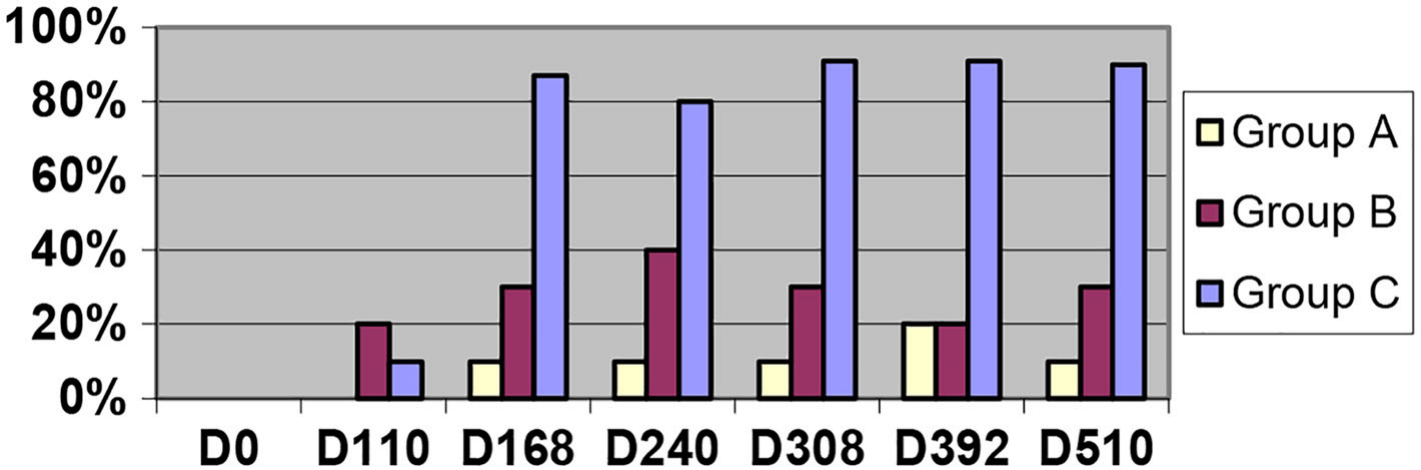
Percentage of cats with enlarged caudal pulmonary arteries on radiographs with score of >2 (scale 0–3) after *D. immitis* infection. Group A (10) Selamectin topical initiated day 28 pi, Group B (10) Oral invermectin initiated day 70 pi, Group C (10) infected no treatment

Cats in Group C had thoracic radiographic scores of >1 in bronchial/interstitial patterns in the majority of cats on Day 110 and for the duration of the study. Pulmonary arterial changes in Group C were consistently noted on Day 168 and consistent for the duration of the study (Fig. 4). The severity (scores 1–3) of the bronchial/interstitial pattern varied in the same cat over time with no identified consistency between cats.

The scoring of the thoracic radiographs noted that the bronchial/interstitial pattern was identified most consistently on Day 168 in Group B cats, and all cats in this group had scores of >1 on at least one of the time points. The bronchial/interstitial pattern in Group B was less severe on Days 308, 392, and 510 with 30% of cats having a score > 2 (Fig. 4). In Group B, enlarged pulmonary arteries were identified in four of 10 cats on Day 240 and then varied within individual cats but was scores were >1 in five of 10 cats at one of the time points of Day 308, 392, or 505 (Figs. 3 and 4). When present, abnormal radiographs in cats in Group B were consistent with lesions in cats in Group C with adult HWs. Radiographic scores of bronchial/interstital changes were significantly different (*p* > 0.05) in Groups B and C compared with Group A. Changes over time were inconsistent within groups for statistical significance.

### Lung histopathology

Evaluation of lung histopathology noted a statistically significant higher score (*p* < 0.01) in Group C cats compared with cats in Groups A and B for all structures examined (Table 4). There was no statistical difference between structures in cats in Groups A and B.

**Table 4:** Histopathologic grading (Scores 0–3) of fixed perfused lung of cats on Day 510 post infection after L3 *D. immitis* infection

Site	Group^a^	Mean Score	Standard Deviation	Significance^b^
Bronchus	A	1.18	0.85	
	B	0.97	0.80	
	C	2.29	0.90	0.001
Bronchiole	A	0.44	0.54	
	B	0.50	0.54	
	C	1.40	0.93	0.001
Alveolus/smooth muscle	A	0.54	0.61	
	B	0.26	0.48	
	C	1.73	1.09	0.001
Arteriole	A	0.80	0.83	
	B	0.80	0.88	
	C	2.18	0.96	0.001
Pulmonary artery	A	0.50	0.54	
	B	0.73	0.81	
	C	2.34	0.84	0.001

^a^Group A (10): selamectin treatment initiated on Day 28 PI; Group B: (10) oral ivermectin treatment initiated Day 70 PI; Group C: infected untreated

^b^Group C (9) was significantly different than Groups A and Group B, which were not significantly different from each other

In evaluation of lung scores, individual cats in Group B (10) had scores of >1 in bronchi (five cats), bronchioles (one cat), pulmonary arteries (two cats), pulmonary arterioles (two cats), and alveolus/smooth muscle (one cat). In Group A cats, scores of >1 in bronchi (five cats), bronchioles (0), pulmonary arteries (one cat), pulmonary arterioles (two cats), and alveolus/smooth muscle (three cats) were noted.

**Fig. 5:**
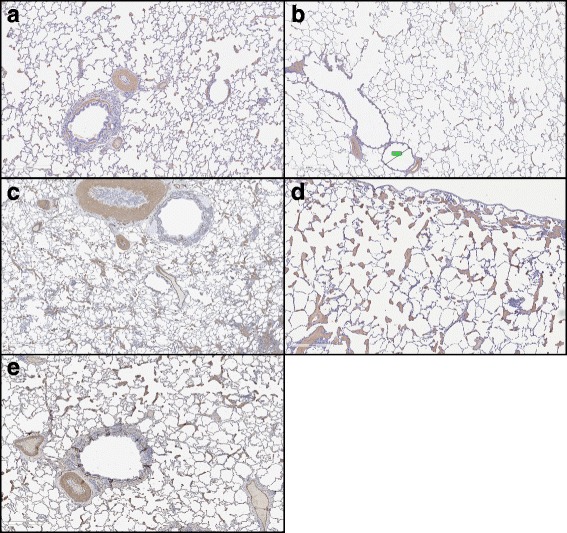
Histopathology of feline lung with alpha smooth muscle actin stain 18 months after L3 *D. immitis* infection. **a** Cats in Group A were treated starting 28 days PI with selamectin. Cats in Group A were generally normal but a wide diversity of abnormal myofibrocyte proliferation was noted between cats and even within the same slide with a subtle increase in isolated area of smooth muscle proliferation, which was interstitial and associated with alveolar struts. In four of the ten cats, isolated pulmonary arterial smooth muscle proliferation was also observed. **b** Cats in Group B had abbreviated immature adult heartworm infections (HARDs) and 18 months PI, some cats had normal areas of lung, but five of the ten cats had multiple areas of smooth muscle proliferation in the interstitium and pulmonary arteries. **c, d**, and **e**, Cats in Group C were infected (untreated) and at 18 months PI had consistent areas of smooth muscle proliferation of the pulmonary arteries and interstitium (**c**). Although present in all groups of cats, the alveolar struts, small pulmonary arterioles, and interstitium proliferation (**d**) was more severe in Group C based on SMA positive myofibrocytes and often in areas not associated with the major pulmonary arteries (**e**). At 18 months PI, cats in Group A and B could not be differentiated by histopathologic examination

Within lung lobes of individual cats in Groups A and B normal lung could be identified in the same section where another airway would be scored as a 2. Further, measure of bronchiole lumen to wall ratio by morphometric analysis did not demonstrate significant differences between groups because of the extreme variability of the lesions within different areas of the same lobe in individual cats in Groups A and B. Individual cats in Group A and B had areas of very marked SMA-positive infiltration in interstitium and periarteriolar areas 18 months after the initial infection (Fig. 5a).

As compared to SPF normal cats, many cats in Group A (infected and treated with preventative medication at 28 days PI) had increased periarterial smooth muscle proliferation and interstitial myofibrocyte infiltration (Fig. 5a). In the Group B (HARDs cats), the changes were less severe than similar changes in the prior 8-month HARD study but were less consistently observed at 18 months (Fig. 5b). Group C cats with adult HWs had more severe smooth muscle proliferation of the interstitial tissues and pulmonary arterial disease with intimal proliferation and smooth muscle hypertrophy were more severe than in Group A and B cats (Fig. 5c). Although present in both Group A and Group B cats, the myofibrocyte proliferation in the interstitium and alveolar struts were most prominent in Group C cats (Fig. 5d). In all three groups of cats, the interstitial and periarterial distribution of smooth muscle actin (SMA)–positive myofibrocytes and smooth muscle was not consistently associated with adjacent bronchial changes (Fig. 5e).

## Discussion

The purpose of this 18-month study was to document the long-term consequences at eight different time points in the pulmonary system of cats when the life cycle of immature adult HWs was abbreviated and no adult HWs developed. Heartworm-Associated Respiratory Disease (HARD) has been demonstrated utilizing this model in a study observed for 8 months PI [2]. The same time points (Days 0, 70, 110, 172, 240) as the 8-month study were utilized and extended in the present 18-month study to include Days 308, 392, and 505. In the 8-month study, the immature adult HWs did not develop into mature adults in cats in the HARD group, HW fragments were not identified, none of the cats became antigen positive, and all developed lung disease not significantly different from cats with mature adult HWs at necropsy [2]. In the present 18-month study, long-term lung pathology was demonstrated resulting from HARD after a one-time single infection. The degree of pathology may be influenced by the number of infective L3 administered as a lower number may have caused fewer changes but the HARD concept is consistent with studies of cats presenting with clinical disease [10–12] and shelter cat studies [13]. This model does not address the clinical consequences in client-owned cats of repeated infection by multiple mosquito bites during mosquito season or yearly reinfection which can be expected in endemic HW areas [3–5, 7, 11].

### Long-term consequences of HARD

In the present study, 18 months after L3 HW infection, pulmonary airway, interstitial, and pulmonary arterial lesions were observed in the cats with abbreviated immature adult HWs (Group B). Based on serial radiographs and histopathology at 18 months PI compared with those at 8 months PI, the HARD lesions became less severe over interval between 8 months and 18 months. Consistent with the results of the 8-month study, HARD was evident on thoracic radiographs of all Group B cats by Day 240. From Days 110 to 240, the majority of Group B and C cats had significant bronchial-interstitial lesions and/or enlarged caudal pulmonary arteries, although Group C cats had pulmonary artery lesion scores consistently higher than Group B cats. Radiographic lesions in Group B and C cats were indistinguishable from each other, were not consistent in each cat for the time points sampled, and were consist with described radiographic findings [16–19]. For each group (A, B, and C), the early (Day 0–240) time points of the earlier 8-month study and this 18-month study had similar changes in the CBC, BAL cytology, radiographs, and serology. These finding confirm that the early pathogenesis of HARD is a dynamic process and is associated with dying immature worms.

Further, the demonstration that the pulmonary intravascular macrophage system is down-regulated in cats with transplanted live adult HWs [20] would suggest a decrease in the inflammatory response in mature live HW infections. These findings would be consistent with the hypothesis that high mortality of immature adults even in cats that develop adult worms is an important cause of HW-induced lung injury.

### Serology in D. Immitis–infected cats

As in the 8-month study, all cats with immature adult HWs were HW antibody positive on at least one time point (Days 110–-240). However, the percent of antibody positive HARD cats from Day 240–510 became lower and only 1 cat was antibody positive on Day 392 and no Group B cats were antibody positive by Day 505. (Figure 1) Cats with mature adult heartworm infection (Group C) remained HW antibody positive for the duration of the observation period. As a clinical assessment of the heartworm status in an individual cat, an HARD cat not developing adult infections should not remain antibody positive 1 year after a positive antibody titer, assuming no subsequent infections. The HW antigen positive result was only associated with mature adult infections and was negative in cats with dying immature adults in the pulmonary arteries. In a client-owned cat which is HW antibody positive with a radiographic bronchial/interstitial lung pattern, a negative HW antibody result at a later date would be consistent with HARD, and this study suggests partial resolution of the disease can be anticipated. But year round heartworm prevention should be maintained to avoid a positive antibody response from pre-cardiac stages after reinfection [15, 16].

### Peripheral CBC and BAL cytology

The changes in peripheral blood eosinophilia (Table 2) and percent eosinophils on BAL cytology (Table 3) in Group B had the same time course response as the heartworm antibody serology. Peripheral eosinophilia is not a correlate of BAL eosinophilic cytology. Peripheral eosinophilia and eosinophilic BAL cytology were most pronounced on Day 110. The number of cats in Group B with abnormal BAL decreased over time and was not observed by Day 505. In Group C cats, after adult heartworms develop, the cat and heartworm would seem to develop a stable equilibrium. Eosinophilic BAL cytology was not a consistent observation in adult heartworm infections and was more associated with the arrival and early death of immature adults. Down regulation of the pulmonary intravascular macrophage system may have a role in the decreased response [20] in mature infections. Early immature pre-cardiac stages of heartworms may initiate a pulmonary response based on eosinophilic BAL cytology in all three groups in this study. Further study has demonstrated an eosinophilic BAL cytology even in cats which had been pretreated with selamectin a month and 2 days before infection [15].

### Radiographic and Histopathologic changes

In comparing the radiographic pattern of Group B cats to Group C cats, the HARD induced by a one-time HW infection did evidence reversibility of some changes in most cats after an extended time period (Day 510). On Days 168 and 240, 7/10 Group B cats had evidence of significant bronchial-interstitial lesions on thoracic radiographs, consistent with HARD induced by immature adult worms, but by Day 308 only 3/10 cats still had radiographic evidence of these lesions. The bronchial-interstitial radiographic pattern was consistent with changes described in asthma, pulmonary fibrosis, *T cati* infection [14], and *Aelurostrongylus* infection [21].

The cats with adult heartworms (Group C) had continued radiographic evidence of HWs day 240–505, however the pulmonary artery enlargement and peribronchial changes were less distinct over time. The decrease in severity of pulmonary radiographic changes over time in cats with mature adults may be a result of the decrease in inflammation as dying worms are eliminated. There is a paucity of long term experimental feline heartworm studies [6, 16, 17, 22]. Serial observation of client owned cats with adult heartworms reported that many cats remain asymptomatic over the course of 4 years before elimination of the infection and eventual death of adult heartworms could be expected to induce acute injury [19].

Lung histopathology also demonstrates that over time, the lung lesions of HARD have some resolution at 18 months PI as compared to the histopathology of the previous 8 month study. The nature of the lesions in the 8 month HARD group was uniform in that most airways were affected to varying degrees allowing for statistical evaluation of bronchial wall to lumen ratios. However, in the 18 month HARD cat lung histopathology, random areas of disease were interspersed with relatively normal areas. In every category scored (bronchus, bronchiole, alveolus/smooth muscle, arteriole, pulmonary artery), Group B cats had significantly lower mean lesion scores than Group C cats. Even in mature adult HW infections (Group C), the lesion scores of bronchi and bronchioles in the cats in the 18-month study were lower when compared with the cats with mature adult HWs in the 8-month study. This is consistent with the hypothesis that early mortality of immature adults induces the significant bronchial and vascular lesions at Day 120 in both cats that develop mature adults and in cats in which no mature adult HWs develop.

### Precardiac D. Immitis–induced changes

Unanticipated findings in this study were observed in the selamectin-treated cats (Group A), which all were HW antibody positive on at least one of the study time points. On Day 110, 50% of the cats were antibody positive and one cat continued to be antibody positive up to Day 308. The elevation of eosinophils on BAL cytology in Group A would suggest a continued pulmonary response to precardiac larvae which was not reflected in the peripheral CBC. On Day 510, the mean lung histopathology scores were not significantly different between Group A and Group B cats, although the areas of interstitial disease were more consistently observed in Group B cats. When HW prevention is introduced 28 days after infection, the exact point in time at which a HW larvae dies and is physically removed is unknown in the cat or the dog. Based on the initial 8-month study and this 18-month study of a single infection, when HW prevention is introduced 28 days PI, precardiac stages of HW larvae survive for an unknown period of time but induce a pulmonary eosinophilic BAL response, positive HW antibody titer, and myofibrocyte proliferation in the interstitial and peribronchial areas and pulmonary arterioles which can be demonstrated 18 months after infection.

A subsequent 8-month study was initiated to address these concerns [15]. Initiation of selamectin 28 and 2 days prior to the L3 infection resulted in none of the cats developing positive HW titers but a very transient eosinophilic BAL response was noted on Day 110 PI. No abnormal radiographs were observed but subtle increases in pulmonary interstitial myofibrocytes of alveolar struts were noted at 8 months PI. Compared with normal SPF cats and cats pretreated before infection [15], cats treated with selamectin on day 28 PI had bronchial, interstitial, and pulmonary arterial histologic changes at both 8 months and 18 months PI. Evidence that soluble products of HWs can induce an innate pulmonary response was supported by interstitial and pulmonary arterial pathology induced by intravenous injection of only the filtered homogenate of HWs for 18 days [23]. Precardiac stages and death of immature *D. immitis* in non-cardiac areas would appear to be associated with a pulmonary reaction as evidenced by the eosinophilic BAL cytology and pulmonary myofibrocyte proliferation. The effects of repeated seasonal infections or yearly reinfections in endemic areas could potentiate this reaction and may be responsible for additional lung injury. The rationale for initiation of HW prevention before mosquito season is reinforced by the present study.

## Conclusion

After the initial phase of HARD in cats from a one-time infection, this 18-month study demonstrates a gradual resolution over time (8 months–18 months PI) based on the lung histopathology and thoracic radiographs and BAL cytology changes over these time points. Randomly distributed histologic evidence of myofibrocyte proliferation was still present, however, 18 months after infection in cats and a bronchial-interstitial lung pattern was evidenced in some HARD cats on radiographic examination at Day 505 PI. The HARD cats continued to have abnormal radiographic lung patterns for months after antibody HW serology and BAL cytology had returned to normal. The acute phase of HARD (Days 110–240 PI) was associated with arrival and death of immature adults 70 to 90 days PI and was clinically indistinguishable from cats with adult mature HW infections. Although there was progressive but incomplete resolution of radiographic bronchial disease by 18 months PI, chronic myofibrocyte proliferation was present at 18 months PI. The resultant restrictive lung disease associated with this myofibrocyte proliferation has been demonstrated by CT analysis in *D. immitis–* and *T. cati–*infected cats [14, 15]. The importance of initiating HW prevention before the mosquito season was highlighted in the selamectin-treated cats (28 days after PI) by the evidence of myofibrocyte proliferation on lung histopathology 18 months PI and the initial eosinophilic BAL cytology and positive heartworm antibody serology associated with precardiac stages of *D. immitis*.

## Abbreviations

Ab: Antibody
Ag: Antigen
BAL: Bronchial-Alveolar Lavage
CBC: Complete Blood Counts
ELISA: Enzyme linked immunosorbent assay
HARD: Heartworm-Associated Respiratory Disease
HW: Heartworm
IM: Intramuscular
IP: Intraperitoneal
IV: Intravenous
PI: Post infection
SMA: Smooth muscle actin
SQ: Subcutaneous
VD: Ventrodorsal
